# p16 Immunostaining of Canine Squamous Cell Carcinomas Is Not Associated with Papillomaviral DNA

**DOI:** 10.1371/journal.pone.0159687

**Published:** 2016-07-21

**Authors:** Silvia Sabattini, Federica Savini, Laura Gallina, Alessandra Scagliarini, Patrizia Bassi, Giuliano Bettini

**Affiliations:** 1 Pathology Division, Department of Veterinary Medical Sciences, University of Bologna, Ozzano Emilia (BO), Italy; 2 Virology Division, Department of Veterinary Medical Sciences, University of Bologna, Ozzano dell’Emilia (BO), Italy; Brigham and Women's Hospital/Harvard Medical School, UNITED STATES

## Abstract

While papillomavirus (PVs) are an established cause of human cancer, few reports have supported a relationship between PV and canine squamous cell carcinomas (SCCs). Human oncogenic PVs lead to an increased expression of the p16 tumor suppressor protein, and the latter can be demonstrated immunohistochemically to support a likely causal relationship between tumor and PV infection. In the present study, archive samples of canine SCC from different anatomical locations were tested by polymerase chain reaction for the presence of PV DNA and by p16 immunohistochemistry. The aims were to investigate the relationship between p16 expression and presence of PV DNA, in order to assess the utility of p16 overexpression as a biomarker of PV infection in canine SCC. A total of 52 SCCs were included. Nine cases (17.3%) showed moderate p16 immunoreactivity, with no association with tumor degree of differentiation, histotype or mitotic activity. The canPVf/FAP64 primers amplified *Canis familiaris* PV-1 DNA from 3 out of 52 tumors (5.8%), one cutaneous, one oral and one tonsillar SCC. There was no association between PV presence and p16 immunostaining. These results do not support a significant role of PVs in the development of canine SCCs. Additionally, PV infection was apparently not the cause of the p16 immunostaining observed in a subset of canine SCCs. A better awareness of p16 level of expression and cellular function in canine cancer may help to define its diagnostic and prognostic role.

## Introduction

Squamous cell carcinomas (SCCs) are malignant tumors arising from the squamous epithelium of skin and mucous membranes. SCCs represent 5% of skin tumors in dogs and are the second most common canine oral neoplasms [1,2]. Less frequent locations include tonsil, nasal cavity, ear canal, mammary gland, lung and anal sac [1,3,4].

The etiology of canine SCC is poorly understood, although environmental factors, including sunlight exposure of lightly pigmented skin, have been associated with the cutaneous form and exposure to urban pollutants may contribute to the development of tonsillar SCC [5,6].

Papillomaviruses (PVs) are small epitheliotropic DNA viruses infecting skin and mucosae. PVs are an established cause of human cancer, and there is accumulating evidence that they may be responsible for the development of a subset of cutaneous SCCs in cats and horses [7,8].

In dogs, 17 PV types have been fully sequenced and classified into three different genera. Naturally occurring PV infections have been demonstrated to cause benign epithelial proliferations, including exophytic or endophytic (inverted) papillomas and pigmented plaques [9]. Papillomas, most frequently occurring in young dogs [10–12], are associated with infection by *Canis familiaris* PV (CPV-1, canine oral PV), CPV-2, CPV-6 and CPV-7. Pigmented plaques, which have been reported to have breed predispositions in pugs and miniature schnauzers, are associated with CPV3-5, CPV-8-12 and CPV-16 [13–15].

To date, few reports have supported a relationship between PV and canine SCC. These include the development of cutaneous SCCs at the site of previous injection with a live CPV-1 vaccine [16] and the malignant transformation of canine papillomas and viral plaques to SCC [12,13,17,18]. Furthermore, papillomaviral DNA has been isolated in several samples of canine mucocutaneous SCCs [6,19–21]. Nevertheless, a causative association, including the interaction of viral oncoproteins with tumor suppressor genes, has never been demonstrated.

Oncogenic human PVs consistently increase the p16^CDKN2A^ tumor suppressor protein (p16), thus the immunohistochemical expression of p16 can be used as a surrogate marker of human PV in head and neck squamous cell carcinoma [22]. An association between p16 immunoreactivity and the presence of PV DNA was observed in feline cutaneous SCCs and different survival times were observed in cats with p16-positive and p16-negative nasal planum SCCs, suggesting that p16 could represent a prognostic indicator [7].

In dogs, only one study on oral SCC has investigated so far the association between p16 expression and PV infection. In that study, despite the finding of p16-positive tumors, no PV DNA was detected, suggesting that the increased p16 was unlikely due to PV infection.

Nevertheless, the biologic behavior of SCC is extremely variable depending on tumour location [23], and different risk factors have been proposed for the different sites, so, possibly, particular forms of SCC may have a distinct etiology. Additionally, as demonstrated in humans, geographic location likely accounts for an important variation in PV prevalence [24].

In the present study, the relationship between p16 expression and presence of PV DNA was investigated on a larger series of canine SCC from different anatomical locations (including anal sac, scrotum, nasal cavity, mammary gland, ear canal and penis). A thorough histopathological examination of the included cases was also performed.

## Materials and Methods

### Ethics Statement

This study is a retrospective investigation carried out on archived tissue samples from canine SCC. As the research did not influence any therapeutic decision, approval by an Ethics Committee was not required.

However, all diagnostic and therapeutic procedures were performed in accordance with the Public Health Service Policy on Humane Care and Use of Laboratory Animals.

All the examined samples were collected for diagnostic purposes as part of routine standard care. Owners gave informed consent to the use of clinical data and stored biological samples for teaching and research purposes.

### Selection criteria and histological evaluation

Histological samples of canine SCC were retrieved from the archives of the Diagnostic Pathology Service of the Department of Veterinary Medical Sciences, University of Bologna, Italy. Breed, age, sex and tumor anatomic location were recorded from specimen submission forms.

Sections (4 μm) of formalin-fixed and paraffin-embedded (FFPE) samples were stained with hematoxylin and eosin (HE) and assessed microscopically by two pathologists (SS, PB) to confirm the original diagnosis. Immunohistochemistry (IHC) for the determination of cytokeratin AE1/AE3 expression was used in the ambiguous cases. Adenosquamous and basosquamous carcinomas were not included. Similarly, the cases warranting malignant pilomatricoma or other follicular tumors as potential differential diagnoses were omitted.

For each enrolled case, the subtype of SCC was assessed according to previously published criteria; conventional SCCs were further graded as well-, moderately- or poorly-differentiated [11,25]. SCCs were classified as well-differentiated when the tumor cells closely resembled normal squamous epithelium, with orderly progression from polyhedral, non-keratinized basal cells to large, polygonal, keratinized cells with evident intercellular bridges and central accumulation of compact laminated keratin (keratin pearls). Moderately-differentiated SCCs showed distinct nuclear pleomorphism, less prominent cell keratinization and rare keratin pearls. Poorly-differentiated SCCs were characterized by a predominance of immature cells, with scant amphophilic or basophilic cytoplasm and absence of intercellular bridges. Keratinization was restricted to single cells and keratin pearls were not observed.

Mitotic activity was also assessed by counting the total number of mitoses in 10 random high-power (400x) fields (HPFs).

### p16 immunohistochemistry

Immunohistochemistry for p16 was performed using a commercial mouse anti-human monoclonal antibody (BD Biosciences, San Jose, CA, USA) whose specificity for canine p16 had been previously validated [21,26].

Endogenous peroxidase activity was blocked by incubation for 15 minutes with 1% hydrogen peroxide in methanol. Antigen retrieval was obtained by incubation in EDTA buffer (pH 9.0) for 20 minutes at 95°C, with a 20-minutes cooldown. Slides were then incubated for 60 minutes with p16 antibody at a dilution of 1:100. Sites of primary antibody binding were identified by incubation with biotinylated goat anti-mouse secondary antibody (Dako, Glostrup, Denmark) at a dilution of 1:200 in blocking solution. Sections were incubated with a commercial streptavidin–biotin–peroxidase kit (Vectastain Elite ABC Kit, Vector Laboratories, Burlingame, California, USA) for 30 minutes; 3,3′-diaminobenzidine was used as chromogen. Sections were counterstained with Papanicolaou’s hematoxylin.

Epithelial cells within the basal layer of epidermis or mucosal surfaces often exhibited weak immunoreactivity and were used as an internal positive control. Negative controls were obtained by omitting the primary antibody.

### Polymerase chain reaction

PCR was performed on FFPE tissue samples. Five-micrometer sections (3 to 5) of paraffin-embedded tumor samples were cut using clean microtome blades and placed directly into PCR tubes to prevent cross-contamination. DNA extraction was performed with NucleoSpin DNA FFPE XS kit (Macherey-Nagel, Germany) with slight modification of the manifacturer’s protocol. Briefly, a maximum of 10 mg of 5 μm thickness tissue section were deparaffinized with 400 μl of paraffin dissolver for 15 minutes at 60°C. Lysis of sample was performed with 100 μl of FL buffer and 20 μl of proteinase K (22 mg/ml) overnight at room temperature. Heat incubation with 100 μl of D-link buffer for each sample eliminated crosslinks from previously released DNA. After the addition of ethanol the lysate samples were applied to the NucleoSpin DNA FFPE XS column, washed and finally eluted in a 20 μl volume of BE eluition buffer. DNA samples extracted were checked for the presence of inhibitors and DNA stability by PCR amplification of 119 bp of the glyceraldehyde 3-phosphate dehydrogenase (GAPDH) gene using the primers dogGAPDHF 5’ GGCGTGAACCACGAGAAGTATAA 3' and dogGAPDHR 5’CCCTCCACGATGCCAAGT 3'. The amplification program consisted in an initial denaturation step of 94°C for 5 minutes, followed by 35 thermal cycles: 94°C for 30 seconds; 58°C for 1 minute; 72°C for 1 minute and a final elongation step 72°C for 5 minutes. To detect PV DNA, three PCR assays with previously published primer pair targeting L1 ORF were performed. The amplification with FAP59/64 primer pair (amplicon length, 478 bp) [27] was performed using the thermal profile of 94°C for 10 min followed by 45 cycles with three steps: 94°C for 30 sec, 50°C for 1 min and 30 sec and 72°C for 1 min and 30 sec, with a final extension time of 72°C for 5 min. The protocol used with the primers combination CanPVf/FAP64, able to amplify 389 bp of the L1 gene, was the same described by Lange and colleagues [28]. The amplification with MY09/11 primers, targeting a 450 bp conserved sequence in the HPV L1 gene, was performed as described by Peyton and colleagues [29]. All PCR reactions have been performed using a no template control and a positive control consisting of DNA extracted from Ca Ski cells (ATCC CRL-1550) and CPV1 DNA that had already been amplified with both FAP59/64 and CanPVf/FAP64 primer pairs.

PCR products were sequenced by Sanger method on both strands of the amplified DNA using an ABI Prism 3100 Genetic Analyzer (Perkin-Elmer Applied Biosystems, Foster City, CA, USA). Chromatograms were analysed using 4Peaks 1.7.1 and sequences aligned and compared by ClustalW implemented in the DNASTAR sequence alignment software (Lasergene, Madison, WI, USA).

### Statistical analysis

Chi-square test and Student’s T test were performed to assess the potential correlations between demographic information, histological features (differentiation, mitotic activity), p16 expression and presence of PV DNA.

Data were analysed by using SPSS statistical software (SPSS, Inc., an IBM Company, Chicago, IL). P values < 0.05 were considered significant.

## Results

The full demographic and histological characteristics of the cases in this study are provided as S1 Table.

### Demographic information

A total of 52 cases fulfilled the inclusion criteria. Fifteen SCCs (28.8%) were in the oral cavity, including dentate jaws (*n* = 10), non-dentate mucosal surfaces (*n* = 1) and tongue (*n* = 4); 10 each (19.2%) were located on the skin and digits, 6 (11.5%) were in the nasal cavity, 5 (9.6%) were tonsillar, 3 (5.8%) were mammary and one tumor each (1.9%) was located in the anal sac, ear canal and penis. There were 24 males and 27 females. The mean (SD) age at the time of diagnosis was 9.8 years (2.7 years). Mixed breed dogs were 14, the most represented purebred dogs included Dalmatian (*n* = 4, all with cutaneous SCCs on the ventral abdomen), Giant Schnauzer (*n* = 4, all with digital SCCs), and German Shepherd (*n* = 4).

### Histological evaluation

Among conventional SCCs, 20 (38.5%) were well-differentiated, 16 (30.8%) were moderately-differentiated and 8 (15.4%) were poorly-differentiated. The remaining tumors were classified as belonging to the verrucous (*n* = 4), papillary (*n* = 2), acantholytic (*n* = 1) and spindle cell (*n* = 1) histotypes. The mean number of mitoses was 28 per 10 HPFs (median, 23.5; range, 3–108). There was no correlation between mitotic activity and tumor location (*P* = 0.846) or differentiation (*P* = 0.354). Cutaneous and digital SCCs were more likely to be well-differentiated compared with tumors arising in other locations (*P* = 0.0031).

### p16 immunohistochemistry

**Fig 1:**
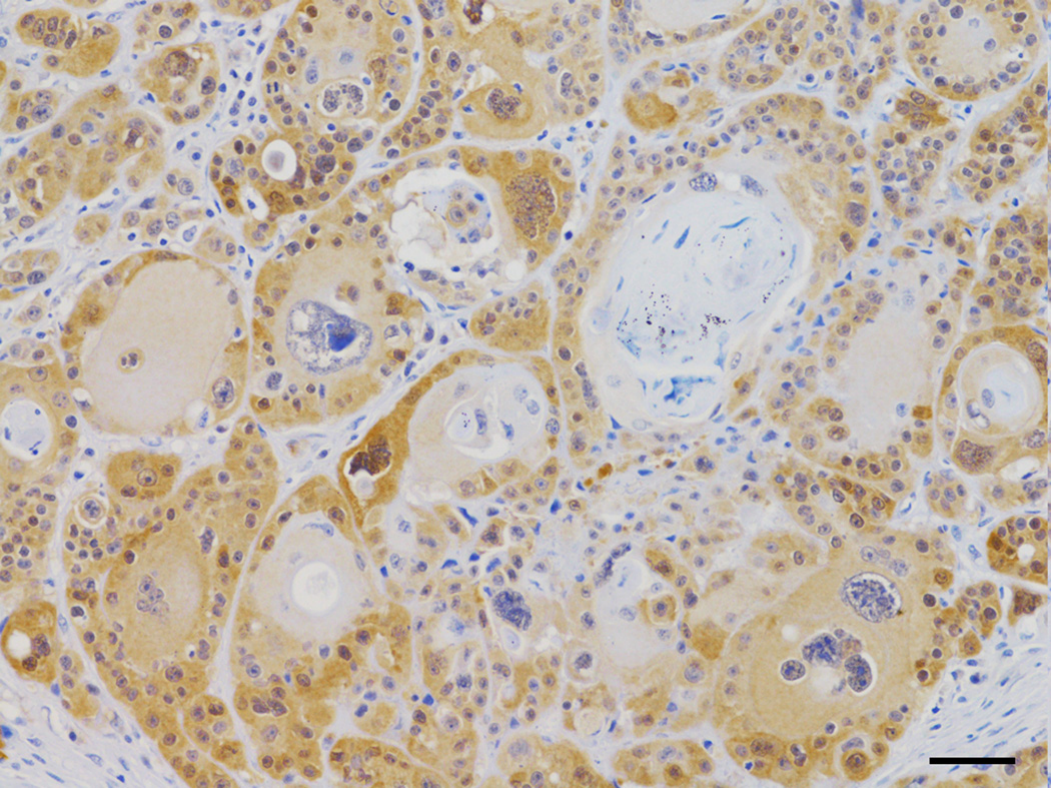
p16 Immunohistochemistry. Dog, squamous cell carcinoma of the scrotum. Moderate cytoplasmic and occasional nuclear immunoreactivity of a part of the neoplastic cells, showing a high degree of anisocytosis and anisokaryosis. Hematoxylin counterstain. Bar, 50 μm.

The p16 antibody labelled tumor cells in 16 cases (30.8%). In 7 (13.5%), immunoreactivity was faint, exclusively cytoplasmic, and restricted to the basal layers of trabeculae, similar to that observed in normal skin. Nine cases (17.3%) displayed moderate to intense cytoplasmic immunoreactivity, with occasional nuclear reinforcement, and were considered p16 positive. Signaling involved also keratinized cells, although staining intensity was stronger in the outer cells. IHC staining involved multifocal spots within the tumor tissue, overall 30–70% of the examined sections. p16 immunoreactive SCCs were located in the nasal cavity (3 out of 6), dentate jaws (2 out of 10), tongue (1 out of 4), digits (1 out of 10) and scrotum (1 out of 2) (Fig 1). Tumor differentiation and mitotic activity were not correlated with p16 immunostaining.

### Polymerase chain reaction

**Fig 2:**
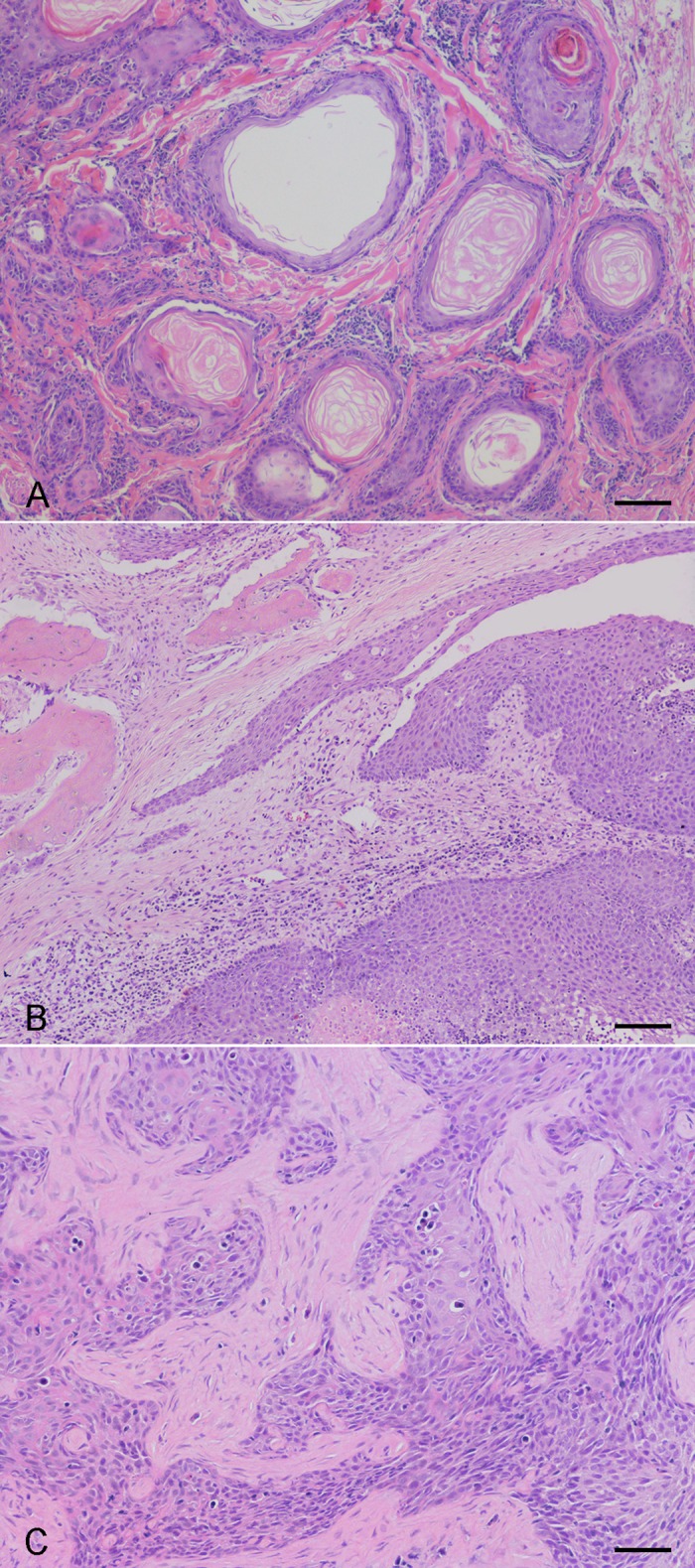
Dog, Squamous Cell Carcinomas (SCCs) with a Positive Canine Papillomavirus 1 (CPV-1) Status. Well-differentiated SCC of the neck region (A), oral papillary SCC (B) and poorly-differentiated tonsillar SCC (C). No prominent signs of viral cytopathology are evident within the neoplasms. Hematoxylin and eosin. Bar, 50 μm (A and C) and 100 μm (B).

All SCC samples tested positive for the GAPDH gene confirming the absence of inhibitors in the DNA samples extracted. The canPVf/FAP64 primers amplified PV DNA from 3 out of 52 cases, one cutaneous, one oral and one tonsillar SCC (Fig 2). FAP59/64 primers were able to detect PV DNA in one of the three canPVf/FAP64 positive samples. No positive case was identified with MY09/11 primers. The sequencing of the three canPVf/FAP64 PCR products demonstrated the presence of Lambdapapillomavirus 2, CPV1. The alignment of the 321 nucleotides of the L1 gene showed a nucleotide identity between 99.1% and 99.4% with the CPV1 (GenBank 9627734) reference strain. Of these tumors, only the oral SCC was characterized by moderate p16 immunoreactivity.

## Discussion

The aims of this study were to investigate the relationship between p16 expression and presence of PV DNA in canine SCC, in order to assess the utility of p16 overexpression as a marker of PV infection in dogs.

Several studies have investigated the relationship between PV and canine SCC. Although some of them identified PV DNA in a subset of cases, the majority of SCCs tested negative. In particular, PV DNA was detected in one eyelid SCC [12], in two dogs with cutaneous SCCs progressing from viral plaques [19], in 4 cutaneous SCCs developed in transplanted dogs with X-linked severe combined immunodeficiency [18], in two of 17 [6] and 1 of 20 cutaneous SCCs [30]. PV-positive oral SCCs were reported in 3 of 29 [20] and 4 of 21 [6] cases. Sequences were consistent with CPV-1, other canine PVs, human PVs or were not identified. Furthermore, a novel PV type (CPV-17) was detected in a dog that developed multiple oral SCCs [21]. Additional studies by Munday and colleagues did not detect PV DNA in any of 23 subungual [31] and 28 oral SCC [26].

In the present study, it was possible to amplify PV DNA from 3 out of 52 canine SCC (5.8%) samples, including one cutaneous, one oral and one tonsillar SCC. In all cases sequences were consistent with CPV-1. The low number of positive cases did not allow statistical considerations about the potential correlation with tumor histotype, differentiation or mitotic activity. Recently, a novel papillary histotype of oral SCC has been described in dogs. This tumor presents with a very similar morphology to that of papillomas, but shows a combination of expansive and infiltrative growth [25,32]. Furthermore, this particular histotype, although potentially occurring in dogs of all ages, is more frequently reported in young dogs, suggesting a hypothetic malignant transformation of an oral papilloma [32]. In the present study, two SCCs were morphologically consistent with the papillary histotype, but it was not possible to amplify PV DNA in either of them.

The infrequent recognition of PV DNA in this and other studies does not support the hypothesis that PVs may represent a relevant cofactor in canine SCC development. However, several novel CPV types have been sequenced recently, suggesting that additional canine PVs may exist [13,33–36]; additionally, PVs from other species, including human PVs, may be inplicated. To increase the chances of detecting known and unknown PV types, three sets of consensus primers were used in the present study. One, CanPVf/FAP64, has been shown to detect five of the seven pathogenic CPVs with high sensitivity [28]. Primer FAP59 ⁄FAP64, were designed to detect human papillomavirus but have been shown to amplify a wide range of PVs from different animal species [37]. Finally, primer MY09⁄11, were designed to amplify PVs from mucosal lesions, in particular human alpha-PV types [38] but were also able to detect canine, feline and bovine papillomaviruses [14,39,40]. When dealing with archive case series, the reliability of DNA analysis and IHC can be negatively affected by former tissue processing [41]. In order to minimize the risk of false negatives, in this study the presence of amplifiable DNA was confirmed within each sample by amplifying part of the GAPDH gene. However, due to fragment length difference between housekeeping gene and the PVs’ amplicons, we cannot fully exclude the presence of viral DNA in some of the GAPDH positive, PV-negative samples.

On the other hand, evidence of PV DNA in neoplastic tissues is not enough to establish a causal association between canine PV and cancer. PV DNA can also be detected in clinically normal skin; therefore, when a PV is found within a SCC, it is difficult to determine whether the PV actually caused the neoplasm or is an ‘innocent bystander’ or a secondary invader of abnormal epithelial cells. Actually, to prove that a PV is directly responsible for the onset of a tumor there needs to be evidence of (1) viral DNA within tumor samples, (2) active viral oncogene transcription in tumor cells, and (3) interaction of viral oncoproteins with tumor suppressor genes [42].

Previous studies in dogs have tried to demonstrate a link between PV and SCC through the immunohistochemical detection of the L1 antigen [43]. This requires active replication of the virus, as this antigen is produced only in the late stages of viral replication [44]. However, the ability of a PV to replicate decreases as epithelial dysplasia increases, and studies of human PV-induced cervical lesions reveal that anti-PV immunostaining is much more common in pre-neoplastic lesions than in invasive neoplasms [45,46]. A similar inverse relationship between cell dysplasia and PV replication seems to occur in feline lesions [7]. In the light of these considerations, the immunohistochemical assessment of L1 antigen appears to be of limited value in evaluating malignant lesions for the presence of papillomavirus.

In human PV-induced head and neck SCCs, degradation of the retinoblastoma protein by the PV E7 protein leads to an upregulation of p16, and the finding of an intense cytoplasmic and nuclear p16 immunostaining in tumor tissue suggests a likely PV etiology [47]. In dogs, preliminary investigations on the relationship between PV infection and p16 immunoreactivity have produced contradictory results. One study reported an intense diffuse p16 immunostaining in a multiple canine oral SCC with papillomaviral DNA and cytopathology [21]. In the same year, a moderate to strong immunostaining for p16 was detected in 4 out of 28 (14.3%) oral SCCs; however, the absence of amplifiable PV DNA in those cases suggested that the increased p16 was unlikely due to PV infection [26].

This is the first study evaluating p16 expression in canine non-oral SCC. According to our results, eight SCCs showed a moderate immunoreactivity, with a stronger staining intensity compared with normal skin and mucosae. However, seven of them did not contain detectible PV DNA. Our data confirm the results obtained by Munday and colleagues [26] and suggest that PV infection may not be the cause of the increased p16 positivity. Moreover, two out of three SCCs that contained amplifiable PV DNA did not show positive immunostaining. This confirms that PVs can be present within a canine SCC without p16 upregulation. This could be due to a bystander infection or to the fact that viral oncogenes do not interact with E7 as in high-risk human PVs.

None of the p16-positive cases in this study showed prominent histological signs of viral cytopathology. The tumor with the highest staining intensity was a scrotal SCC showing marked characters of cellular atypia, including prominent multinucleation and presence of giant cells. This histological appearance is usually not attributed to viral cytopathology and is in contrast with that reported in human PV-associated SCC, typically showing a basaloid, low-grade morphology [48]. Alternatively, these features could simply reflect a higher malignancy, which was confirmed in that case by the remarkably high number of mitoses (71 in 10 HPFs).

Plenty of studies are available about the downregulation of p16 as a tumor suppressor protein in cancer, but limited information is available regarding its overexpression in non-HPV related tumors [49–52]. In humans, it has been hypothesized that p16 overexpression in tumors can be indicative of two main situations: in benign or pre-malignant lesions, p16 may be overexpressed as a consequence of oncogene-induced senescence, whereas in malignant lesions, overexpression appears to be a mechanism to arrest the uncontrolled proliferation caused by failure of the Rb pathway (secondary in this case not to viral infection, but rather to mutational silencing of the Rb gene) [52].

Unfortunately, a relationship between p16 immunostaining and tumor biologic behavior was not assessed in this study due to its retrospective nature and limited number of cases.

A better understanding of p16 overexpression in oncology may suggest new possible applications of p16 as a diagnostic or prognostic marker. At last, because p16 is one of the most important tumor suppressor proteins, new anticancer therapies involving restoration of its functionality could be investigated [52].

## Conclusions

In the present study, PV DNA was only detected in three of 52 samples of canine SCC. These results do not support a significant role of PVs in the development of canine SCCs. Additionally, in contrast to human and feline SCC, PV infection does not seem to be related with p16 immunostaining, which was observed in nearly 20% of cases. A better awareness of p16 level of expression and cellular function in canine cancer may help to define its diagnostic and prognostic role and to guide research on potential therapies.

## Supporting Information

S1 Table Supplementary data. Demographic and histological characteristics of the 52 cases of squamous cell carcinomas in this study.
(XLSX)
